# Digital Health Technologies for Maternal and Child Health in Africa and Other Low- and Middle-Income Countries: Cross-disciplinary Scoping Review With Stakeholder Consultation

**DOI:** 10.2196/42161

**Published:** 2023-04-07

**Authors:** Sarina Till, Mirriam Mkhize, Jaydon Farao, Londiwe Deborah Shandu, Livhuwani Muthelo, Toshka Lauren Coleman, Masenyani Mbombi, Mamara Bopape, Sonja Klingberg, Alastair van Heerden, Tebogo Mothiba, Melissa Densmore, Nervo Xavier Verdezoto Dias, CoMaCH Network

**Affiliations:** 1 School of Information Technology Independent Institute of Education Durban South Africa; 2 Department of Computer Science University of Cape Town Cape Town South Africa; 3 Human Sciences Research Council Centre for Community Based Research Sweet Waters South Africa; 4 Faculty of Health Sciences University of Limpopo Polokwane South Africa; 5 South African Medical Research Council/Wits Developmental Pathways for Health Research Unit University of the Witwatersrand Johannesburg South Africa; 6 Department of Computer Science Cardiff University Cardiff United Kingdom; 7 See Acknowledgments

**Keywords:** maternal health, child health, digital health, community, scoping review, low- and middle-income countries, LMIC, technology, co-design, mobile phone

## Abstract

**Background:**

Maternal and child health (MCH) is a global health concern, especially impacting low- and middle-income countries (LMIC). Digital health technologies are creating opportunities to address the social determinants of MCH by facilitating access to information and providing other forms of support throughout the maternity journey. Previous reviews in different disciplines have synthesized digital health intervention outcomes in LMIC. However, contributions in this space are scattered across publications in different disciplines and lack coherence in what digital MCH means across fields.

**Objective:**

This cross-disciplinary scoping review synthesized the existing published literature in 3 major disciplines on the use of digital health interventions for MCH in LMIC, with a particular focus on sub-Saharan Africa.

**Methods:**

We conducted a scoping review using the 6-stage framework by Arksey and O’Malley across 3 disciplines, including public health, social sciences applied to health, and human-computer interaction research in health care. We searched the following databases: Scopus, PubMed, Google Scholar, ACM Digital Library, IEEE Xplore, Web of Science, and PLOS. A stakeholder consultation was undertaken to inform and validate the review.

**Results:**

During the search, 284 peer-reviewed articles were identified. After removing 41 duplicates, 141 articles met our inclusion criteria: 34 from social sciences applied to health, 58 from public health, and 49 from human-computer interaction research in health care. These articles were then tagged (labeled) by 3 researchers using a custom data extraction framework to obtain the findings. First, the scope of digital MCH was found to target health education (eg, breastfeeding and child nutrition), care and follow-up of health service use (to support community health workers), maternal mental health, and nutritional and health outcomes. These interventions included mobile apps, SMS text messaging, voice messaging, web-based applications, social media, movies and videos, and wearable or sensor-based devices. Second, we highlight key challenges: little attention has been given to understanding the lived experiences of the communities; key role players (eg, fathers, grandparents, and other family members) are often excluded; and many studies are designed considering nuclear families that do not represent the family structures of the local cultures.

**Conclusions:**

Digital MCH has shown steady growth in Africa and other LMIC settings. Unfortunately, the role of the community was negligible, as these interventions often do not include communities early and inclusively enough in the design process. We highlight key opportunities and sociotechnical challenges for digital MCH in LMIC, such as more affordable mobile data; better access to smartphones and wearable technologies; and the rise of custom-developed, culturally appropriate apps that are more suited to low-literacy users. We also focus on barriers such as an overreliance on text-based communications and the difficulty of MCH research and design to inform and translate into policy.

## Introduction

### Current State of Health Care and Information and Communication Technologies for Health (Digital Health) in Low- and Middle-Income Countries

Low- and middle-income countries (LMIC) have a long history of poor access to and poor quality of health services, particularly in the maternal and child health (MCH) arena [1-4]. The major issues impacting the quality of MCH services include the lack of human and physical infrastructure owing to the unavailability of safe clinical facilities, diagnostic equipment, and medication resources as well as a lack of training programs for health care workers. All these issues are leading to low quality of care, inadequate diagnosis and treatment, high infant mortality rates [5,6], a high number of infections during pregnancy, an increased risk of mother-to-child HIV transmission, an increased risk of malnutrition, early childhood pneumonia, and many other health-related issues [5-19]. Health inequalities in early life can perpetuate lifelong social inequalities if not addressed and mitigated.

The increased use of information and communication technologies (ICTs) for health, termed as digital health [20], has shown promise in supporting MCH in LMIC by facilitating women’s access to and communication with health services as well as supporting health care professional care practices, treatment, and diagnosis [21-23]. While the digital transformation in health care is opening several opportunities for improving MCH outcomes by supporting service users and health care providers alike [20] and promoting healthy behaviors during pregnancy in high-income countries [24], the transformation continues to be a challenge with regard to designing digital health technologies for low-resource settings in LMIC [25,26], and we know little about how digital health technologies work and for whom in the right context. In particular, most of these technologies have been implemented to promote the societal and global health ideals of motherhood and overlook the social and cultural practices of local communities that often do not have a voice in the design process, producing a narrow view of the local context of the beneficiary populations [20].

Existing communication barriers, power imbalances, conflicts, and distrust among multiple stakeholders [26,27] are a few examples of digital health consequences when failing to account for the sociocultural contexts and realities of the communities and their participation in the design process. Digital health can certainly play a role in addressing maternal health challenges, but it can also exacerbate inequalities if not done in a contextually relevant manner. Therefore, it is not surprising that current approaches to digital MCH are failing because of design practices not being situated within the realities of the community and a lack of meaningful involvement of members of the community in the design process [28,29].

Sociotechnical approaches to digital health technology design and implementation exist, such as community-based co-design, and are important to consider to meaningfully engage with community participants and increase the impact of health interventions [30,31]. Co-design approaches harness health care professionals’ creativity as well as the creativity of people who have firsthand knowledge of the problems they experience [26]. Although many definitions of co-design exist across disciplines, for this study, we drew on several sources in the literature to build the following definition:

Community based co-design is a design method that is conducted with and within the community [32] to enable the participation and meaningful engagement which respects the values and cultures of that community [33]. This method provides communities with an equal voice and stake in the design process and brings community members in dialogue together with other project stakeholders as equal contributors to the design and deployment process [34]. This is done in order to develop interventions with a higher likelihood of being beneficial to the community.

### Scoping Review Rationale and Study Overview

Therefore, it is important to synthesize and better understand the role of community-based co-design approaches in the design and implementation of digital MCH interventions. While previous reviews focusing on digital health intervention outcomes in LMIC are available across different disciplines [26,35-37], there is a lack of coherence with regard to what digital maternal health means across these fields. Cross-disciplinary reviews are becoming increasingly important [38] for providing a holistic understanding across fields. However, there are few attempts that consolidate learning from across different disciplines in the context of digital health technologies but are limited in relation to MCH in LMIC. In May 2020, we started a project titled “Co-designing Community-Based Information and Communication Technology Interventions to Enhance Maternal and Child Health in South Africa (COMACH),” aimed at developing and consolidating a cross-disciplinary network (local and international partners, researchers, academics, and community leaders), understanding current research and practice, and working with community stakeholders to determine their priorities for maternal and child health and well-being by exploring innovative ways to address the identified MCH priorities through digital health in South Africa. Leveraging the diversity of our interdisciplinary project and the COVID-19 pandemic restrictions, our first step was to conduct a scoping review with stakeholder consultation to outline existing research on MCH interventions, including the co-design of community-based ICT health interventions, to inform our future research agenda. In contrast to previous reviews [26,35-37], we conducted a cross-disciplinary scoping review that included the experiences and work of researchers from 3 major disciplines to provide a more holistic understanding across fields on the current state of digital maternal health technologies: public health, social sciences applied to health, and human-computer interaction (HCI) in health care and among local practitioners (eg, nongovernmental organizations [NGOs]). Scoping reviews are popular because this method synthesizes research evidence and maps the body of work in an existing field in terms of volume, nature, and characteristics of the primary research [39]. In the following sections, we present our methods, followed by our review findings and discussion, outlining the key challenges and opportunities across these disciplines.

## Methods

### Overview

**Figure 1:**
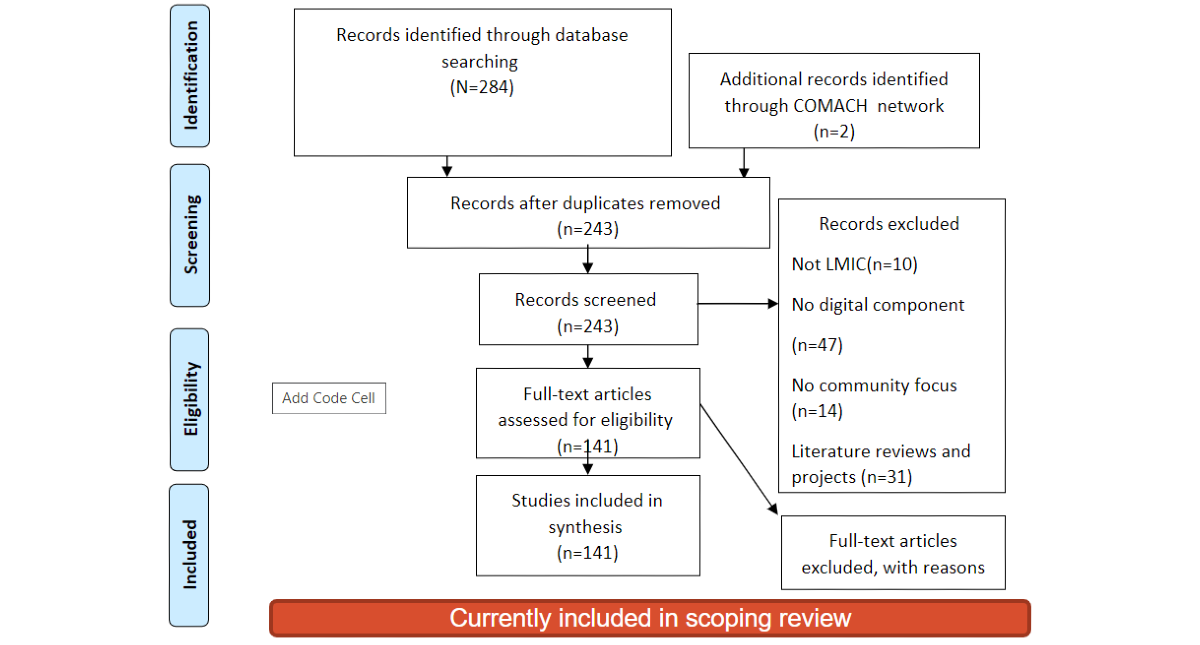
PRISMA (Preferred Reporting Items for Systematic Reviews and Meta-Analyses) diagram. COMACH: Co-designing Community-Based Information and Communication Technology Interventions to Enhance Maternal and Child Health in South Africa; LMIC: low- and middle-income countries.

We framed this scoping review using the Arksey and O’Malley [40] scoping review framework, consisting of a 6-stage methodology (identifying the research questions, searching and identifying the relevant studies, study selection, charting the data, summarizing and reporting on the data, and finally an optional consultation with stakeholders) for conducting scoping reviews. Our process included the following five steps: (1) identifying the research question, (2) searching and identifying the relevant literature and projects, (3) study selection through preliminary tagging and analysis, (4) consultation with stakeholders, and (5) consolidating the literature review and interview results. Finally, we included a table for Preferred Reporting Items for Systematic Reviews and Meta-Analyses (PRISMA) guidelines to illustrate the scoping review findings (Figure 1). To inform and validate the review [41], we conducted interviews with key stakeholders (optional stage in the Arksey and O’Malley framework [40]) who have expertise in MCH, taking advantage of interdisciplinary coinvestigators as well as local and international partners, researchers, academics, community leaders, and facilitators within our project network in COMACH. In addition, a scoping review reporting on scoping reviews showed how only a small proportion of reviews have included the stakeholder’s consultation stage and highlighted their importance to ensure that the review findings are relevant to the context of research and practice [42].

### Step 1: Research Question

Aligned with recent methodological guidance for scoping reviews [43], we aimed to answer 1 primary research question for this review in the context of digital maternal health interventions in LMIC:

- To what extent do digital MCH projects in LMIC engage the communities? Who is involved; how are they involved; and when are community members included in the design and implementation process?

### Step 2: Searching and Identifying Relevant Literature and Projects

#### Inclusion and Exclusion Criteria and Paper Selection

This review aims to provide a cross-disciplinary perspective on the literature reporting primary research in community-based digital MCH interventions in LMIC. Thus, we only included articles in the review that (1) contained a community focus, (2) took a co-design approach, and (3) had digital deployment. Aligned with a recent scoping review [44] and methodological guidance [43], we excluded publications without primary research such as editorials, research protocols, short abstracts, reviews (secondary research), and publications without digital deployment and not reported in English. Three researchers from the fields of public health, HCI, and social sciences applied to health used a curated set of search terms (Multimedia Appendix 1) to identify the literature for their discipline in the following web-based databases: Scopus, PubMed, Google Scholar, ACM Digital Library, IEEE Xplore, Web of Science, and PLOS. We used the search terms and identified 284 articles with duplicates, as ≥1 researcher identified some articles. We also considered unpublished gray literature recommended by members of our research network and other researchers, such as websites for MCH projects that have not been formally researched.

#### Data Extraction and Classification Framework

Next, we created a data extraction and classification framework to support preliminary tagging (see the following section) and subsequently excluded articles from the review. The research team met online and brainstormed different characteristics based on the content and keywords of the articles to be included using a collaborative tool on the web (Jamboard [Google]). For each paper in the review, we extracted 10 characteristics including caregivers, values embedded in the design of the technology, the technology used, context, deployment, measure, implications, methods, target, and stakeholders. With these characteristics, we inquired what technology was deployed, what the articles evaluated, were there any implications for policy makers, who were the target group or users, and who were the stakeholders involved in the research. Each characteristic also has subcharacteristics; for example, the “Caregiver” tag was subtagged with “father,” “single parent,” or other tags that indicate who was explicitly included in the paper.

### Step 3: Preliminary Tagging and Paper Analysis

#### Overview

We then discussed the tags that were not agreed upon and ultimately decided on each paper’s set of tags. Following this process helped us ensure that at least 1 other researcher verified each paper’s set of tags. Papers that did not fully meet the inclusion criteria were moved to a different collection within Zotero, a reference management software system that we used to facilitate the analysis [45].

#### Tagging and Analysis

Each researcher independently screened titles and abstracts to identify full-text articles to be included in the review. Then, 3 researchers tagged all the articles sourced for their discipline and added their initials to indicate that they were the researchers who created the initial set of tags and to confirm that they agreed on each other’s tags. All tags that had a full set of 3 initials were annotated with an asterisk (*), indicating that these tags were final and accepted. All tags with 1 or 2 sets of initials were then discussed and either adopted or dropped.

The scoping review table recommended by Arksey and O’Malley [40] was adapted to summarize the content of the articles (Multimedia Appendix 2 [26,40,46-139]) to facilitate the reviewing process. Each researcher was responsible for summarizing the articles in their respective disciplines using headings such as Author, Objective, Topic, Design, Sample, Technology used, Deployment duration, Intervention description, Outcomes and Measurement, and Results and Recommendations.

Summaries were created for the remaining 141 articles in the 3 disciplines and used for analysis, which we discuss in the *Results* section.

### Step 4: Interviews With Key Informants—Stakeholder Consultation

#### Participant Recruitment

Following the optional stage from the scoping review framework [40], we conducted stakeholder consultation by conducting interviews with key expert informants to inform and validate the findings of the review to ensure they are relevant to the context of research and help us define our research agenda. Although very few scoping reviews have consulted stakeholders, this is an important step in ensuring knowledge translation [42]. Thus, we used purposeful sampling [140] and recruited participants who were aged ≥18 years with expertise in MCH within our research network, including cross-disciplinary researchers from social sciences applied to health, HCI, and public health as well as NGOs, community facilitators, and international interdisciplinary collaborators. Unfortunately, owing to the COVID-19 pandemic restrictions, we could not include community members at this stage. We have provided limited demographic information (due to ethical clearance) about our stakeholders in Multimedia Appendix 3.

#### Interview Guide

The interdisciplinary nature of this study had the potential to introduce inconsistencies in interviews and subsequent interview data. Thus, we decided to use a standardized interview guide informed by the preliminary findings from the scoping review. This script investigated the following: (1) the values and definitions related to community-based co-design; (2) details regarding current research projects related to COMACH; (3) the challenges researchers faced while conducting research in terms of ICT, engaging with and accessing their research communities; and (4) best practices in terms of ethical considerations, mainly when working in MCH. The aforementioned ethical considerations included the management of power dynamics, engaging the community in research activities, and finally the most appropriate and successful methods in terms of community-based co-design with a particular focus on MCH. The script was pretested with the interviewers and the project management team, and researchers were required to practice interviews with one another before conducting the scoping review interviews. The interview guide is provided in Multimedia Appendix 4.

#### Interview Process

We chose web-based interviews to accommodate both the geographic distances between the participants and the various COVID-19 pandemic lockdown restrictions. Between June and October 2020, 3 researchers conducted the interviews in English to accommodate all the researchers who would assist with the analysis of the interview data.

We emailed 34 participants invitations to schedule a 45- to 60-minute interview at their convenience using a web-based diary management system. This invitation contained the participant information sheet, informed consent form, and review table containing all the literature we gathered. This email further instructed the interviewees to return a signed informed consent form to the researcher before the interview. In total, 28 participants responded and participated in the interview process; 6 represented social sciences applied to health, 7 represented public health, 6 were practitioners (eg, community facilitators and NGO personnel) and 9 represented HCI research in health care.

Each interview started with the researcher introducing themselves and ensuring that the informed consent form was received and signed by the participant. The researcher next asked permission to record the interviews. The researcher then conducted the semistructured interview using the interview guide (Multimedia Appendix 2). We uploaded the interview recordings to an adequately secured data cloud that only we and 3 network partners could access. Next, we electronically transcribed the recordings. Each transcription was anonymized to not include any information that could identify the participants.

#### Coding Interviews and Thematic Analysis

We analyzed our data using a codebook thematic analysis [141,142] and used NVivo (QSR International) qualitative data analysis software to support the analysis and stored the NVivo project on an adequately secured data cloud. Five researchers met after all the interviews were conducted and collaborated to create a shared codebook based on the tags and subtags used to analyze the literature. We then used the codebook to analyze the interview data. This process was followed to consolidate the literature and interview data to obtain consolidated findings.

We remained in constant communication to discuss any new codes that emerged during the coding process. New codes were shared to include the code in their respective codebooks to ensure that all of us had access to the same codes. Finally, we met online once all the coding processes had been completed and verified the assigned codes to ensure that the data were correctly coded. The researchers agreed on the various themes and subthemes and combined them into 1 consolidated document. The themes were then shared with the research network for final comments and recommendations. This iterative process, which leveraged open communication, was followed to ensure the validity of our analysis [141].

### Step 5: Consolidation of the Literature Review and Interview Results

One researcher consolidated the interview and literature review data. This was possible because our interview codebook was based on tags and subtags used to categorize the literature identified through our database searchers. Therefore, this study combined the literature and interview data according to the tags and codebook and derived the overlapping themes, which we have discussed in the subsequent section.

### Step 6: PRISMA Diagram

Our systematic review process is outlined in the PRISMA diagram in Figure 1.

### Ethics Approval

Ethics approval was obtained from the University of Cape Town in South Africa (reference number FSREC 040-2020) and confirmed by Cardiff University’s ethics committee who accepted the approval from the University of Cape Town.

## Results

### Overview

From a sample of 243 articles, we excluded a total of 71 articles from the scoping review on the basis of not being related to MCH (n=47), not being related to mobile health (mHealth; n=14), and not being within the scope of LMIC and Africa (n=10). Finally, a total of 141 articles were included in the scoping review across disciplines, including 49 articles from the HCI community, 58 articles from public health, and 34 articles from social sciences applied to health. The articles predominantly focused on health education [46-49]; staff training and e-learning [50]; breastfeeding [51]; child nutrition [26,51-55,143]; health care; and follow-up on health services by community health workers (CHWs) [27,57,58], maternal health [59-61], and maternal mental health [19,58,62-64,144]. There is still a large focus on the digitization of record keeping, especially for the tracking of immunizations [58,65]. We present the major themes from these articles, along with our interview data, in the following sections.

### Values Identified

Our first area of interest was the values that researchers attach to community-based co-design. We found that very few articles directly discussed the values embedded in the technology or any values underpinning the studies conducted by researchers. However, we extracted the following values from the literature: (1) empowerment (n=3) by encouraging the participation of mothers to unpack neonatal intensive care unit (NICU) communications [26] and, through positive enforcement, to promote the donation of donor breast milk [56]; (2) input and feedback (n=5) by involving parents on voice messages regarding young child feeding practices [66] and CHWs on mHealth apps developed for them [67]; (3) empathy (n=1) when designing digital solutions for mothers with babies in the NICU [56]; (4) sustainability (n=1) by advocating that mHealth studies move out of the exploration phase to be more sustainable [68]; and (5) finally, creativity and inclusion (n=3) that included asking community members to evaluate video content that encourages exclusive breastfeeding (EBF) [78] and encouraging parents to record and create their own video content to support MCH practices [62].

**Figure 2:**
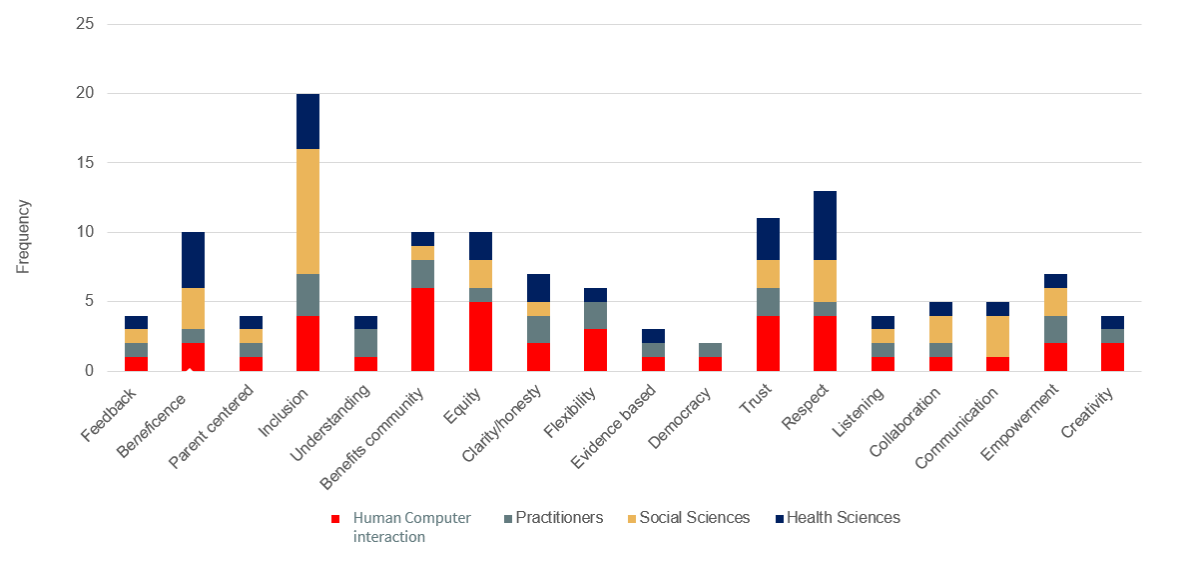
Values identified during interviews by the practitioners and the interdisciplinary researchers.

These findings contrast with our *interview* data, which provided rich insights into researchers’ and practitioners’ values attached to their work. Figure 2 shows a breakdown of the values listed per discipline during our interviews.

The most mentioned value varied by discipline and practice with the HCI research in health care participants mentioning “benefitting the community” followed by “equity” and then by “trust” and “respect,” which received equal mentions. For example, 1 researcher expressed the following:

I think the biggest value I would say is honesty in the sense of expressing and explication of intentions of why are we doing what we’re doing and as well as sharing a bit of vulnerability as an opening up for others to be honest about. Why are they involved in the project? What are their motivations?

HCI researcher

The social sciences applied to health researchers attached great value to “inclusion,” with all of them mentioning this value, followed by “beneficence” and “respect.” For example, 1 researcher stated the following:

Ideally, you’d want to get communities involved in the very design stage before you apply for funding so that they can feed in more directly what they want.

Social sciences applied to health researcher

The public health scientists mentioned “beneficence” the most, followed by “respect” and “inclusion,” and finally the NGO practitioners mentioned “inclusion” most, followed by “trust” and “equity,” with 1 researcher stating the following:

So, the value is that there must be mutual respect between researchers and stakeholders there must be also a mutual benefit, not just respect so that there should be benefit out of it. It should be value added. In other words, the outcomes must get some tangible value to the community

Public health researcher

OK, so I think it is it collaborative would be the first one and respectful as well, so understanding that there are sometimes power dynamics involved with it.

Practitioner

While there is a diverse list of values spread across disciplines, it is also noteworthy that inclusion, trust, and respect are common to and highly valued in all disciplines.

### Technologies Leveraged

We then explored how MCH researchers leveraged ICT (Table 1). This review highlights 92 articles with a digital intervention. These interventions take the form of (1) SMS text messages (text messages sent using the SMS) and platforms (n=29, 32%) [49,65,69-72]; (2) custom-developed mobile apps (tailor-made interventions that can include images, video text, and voice; n=42, 46%); (3) web-based applications (n=6, 7%); (4) social media such as Facebook, Twitter, and WhatsApp (n=6, 7%); (5) movies, videos, and voice messages (n=4, 4%); and finally, (6) sensory data collection from Bluetooth and wearable technology (n=5, 5%) [62,63,74,77,143,145,146] that are currently deployed in and dominate the MCH arena in Africa and other LMIC. The literature further highlighted the value of SMS text message deployment in terms of accessibility and cost [49,56,65,70,71,73]. Voice technologies are recommended for low-literacy settings [35]. Custom-developed app interventions are currently the predominant deployment method, with 42 studies implementing this technology for their interventions. Unfortunately, there is still little innovation present, with only 7 articles using video content and sensor-based data derived from wearables and newer methodologies such as gamification.

**Table 1:** Percentages and type of technologies deployed in the literature.

Technologies deployed	Article (n=92), n %
Custom-developed mobile apps	42 (46)
SMS text messaging	29 (32)
Web-based applications	6 (7)
Social media	6 (7)
Wearables	5 (5)
Videos, voice messages, and movies	4 (4)

In contrast to the literature, the *interview data* highlight more innovation with interventions by providing examples such as automated reply services aimed at assisting new parents with questions and doubts and implementing WhatsApp to give women living in patriarchal societies a better chance of accessing health information. A researcher offered the following example:

We also saw WhatsApp being used from women to women and also from the community health workers to woman and also from community health work, some community health workers to discuss many different cases to call for help. Um? To ask for, I don’t know if they needed to do a follow up check-up or need to go to the hospital. They were using WhatsApp a lot. There is another tool that they were using in India that is similar to do too, but it’s the same functionality or trying to look for videos and stuff, yeah? Um?

HCI researcher

Another use of WhatsApp is health education through animated video content shared via WhatsApp messaging, as detailed by another researcher:

We worked quite hard to keep WhatsApp messaging with. The district health authorities, even when we weren’t in the field back in, you know, March, April, May, June, time so that we didn’t lose the momentum we gained in meeting the community back in January and February. So, Technologies played a role there, so although we can’t use technology with our participants in the Maasai communities, we can use it with the leaders ‘cause they do have access to technology.

Public health researcher

### Community Involvement in Existing MCH Projects

Next, we inquired about how and when existing LMIC projects include communities in the design and deployment of their projects. We found that this is done in various ways, ranging from including the community from the start of the design process by making use of initial user-centered design, using co-design [56], and finally using usability studies after the project had been launched [49,65]. We further found that there is a definite lack of community engagement and formative research to ensure that cultural traditions are considered [60,147]. Many studies have used expert-led design that did not include the community [28,75,148,149]. Unfortunately, this led to at least 1 MCH project failing because it did not consider current community realities. In addition, this particular project did not consider mobile phone ownership and the cost of SMS text messages and ultimately had low uptake and limited reach [28]. Our interview data echo these findings, with some of the researchers stating that they include the community from the very beginning of a project as equal design partners and experts:

They were the experts basically. So, we have information from the literature how diabetes works without the factors. But then when? Kind of confronted with their life left experiences and with their practices. Yeah, they helped us to clarify and all our assumptions, they ruined our assumptions.

HCI researcher

In contrast to the previous statements, 1 researcher mentioned that they do not really involve the community in the design, only as evaluators:

Haven’t really involved codesign. They’ve involved evaluation of certain interventions or looking at the extent of the health problem

Public health researcher

### Differences in Understanding and Lacking a Common Language Regarding Community-Based Co-design

The scoping review data further emphasizes that the definition of community engagement and co-design varies according to discipline and between researchers within a particular discipline. For example, at least 1 participant in each discipline made no distinction between community engagement and community-based co-design, with some participants admitting that they did not understand the difference:

OK so I have to admit I don’t understand exactly the difference.

Social sciences applied to health researcher

I don’t know. I can’t really see a difference between them. OK, to be honest, because either way the communities involved, it’s a gift, a thing, so whatever information that they provide to you is also information that although I mean there’s also give back, you know from the start. So yeah, I mean engagement is a bilateral process.

Public health researcher

OK. I don’t think there is. I don’t think there’s a major distinction between the two. I think like. You know community engagement. If you’re engaging with the community, you know presumably, your there to just sort of seek knowledge and trying to understand what you’re aiming to sort of do within that within that community.

HCI researcher

The public health sciences had the most participants who could not or did not make a clear distinction between community-based co-design and community engagement, with 2 researchers stating:

Well, maybe Codesign is about a product or a resource or a[n] ICT kind of digital thing, whereas community engagement could be about anything.

Public health researcher

You can’t really make a distinction because both of them you involve the community in the interventions that he wants to bring about... You engage the community to come up with solutions to their health problems. OK.

Public health researcher

While the social sciences applied to health provided differences in the definitions, none of them touched on the fact that community-based co-design aims to see the community as equal design partners who are experts in the context of the problems they are experiencing. It should be noted that HCI-in-health-research participants provided well-known definitions that focused on the importance or the relationship with the community and the need for lower power differences. However, only 2 researchers mentioned equal design partners, and 1 researcher stated the following:

But I think the fact is that if we aren’t actually asking them to be a part of that design, we’re missing out on all of the really interesting approaches that we could never think of...Unless we have somebody else thinking of them right, and so if we are seeking to really disrupt the current trajectories of you know, learning health, seeking health care, all of this and maternal and child health, then one of those places where we can find disruptive innovation is from within the community. From that perspective, its mind boggling that we expect that the ideas for disruptive innovation to come from only Steve Jobs, right?

HCI researcher

### Who Have Been Involved—Stakeholders Identified

Next, we considered the community members who were included in the literature and community projects. The literature references mothers (n=80), CHWs (n=28), single parents (n=16), fathers (n=13), grandparents (n=4), and others (n=2). This review further considers that the MCH arena often includes mothers as caregivers and in an implicit manner in the literature. Surprisingly, the caregivers targeted in most studies are not explicitly mentioned. In this review, we found that most articles (n=28) focused on CHWs as service providers’ health education and information [76,150] related to nutrition [34,51,78-80], mental health [81], and other health-related practices. CHWs are also seen as users of apps [47,82,83,150] and web-based platforms [12,48,63], social media [84,146], and digital sensory data [144] aimed at supporting CHWs in service delivery. Moreover, our interview data indicated that CHWs are valued in the co-design process or at the very least consulted in the design process:

You know, helping us like enhance the product in itself, which obviously then goes on to benefit the next set of Community health workers you know in terms of accessing a product and a service that’s informed by a range of Community health workers who continue to give us like feedback. And so yeah, I think that from a technology perspective, I think the communities have played a huge role in sort of both enhancing the specific application and then helping us Co-Design and Co-Create also indirectly enhance the product in many ways over the years.

Practitioner

We further found that fathers are underrepresented with only 11 articles mentioning or targeting fathers. Most of these articles also focused on nutritional outcomes and infant health [51,52,57,60,64,66,86-88,144]. Only 2 articles referred to fathers in terms of child development [57,88]. Thus, fathers are often excluded in other areas of MCH. This finding was supported by interview data, with 1 researcher stating the following:

The challenges that I had was to having, for example more fathers, more men involved. But it was easier to have mothers and in this kind of issues then to have fathers involved and giving feedback and actively play a role.

HCI researcher

This researcher further explains that fathers often feel out of place in the MCH space because the existing resources are not tailored to and do not target fathers:

Even when fathers tried to take part of it, they feel that they do not belong there, for example, fathers has completely different ways to interact. If you are a mother of if you are father and we have specific content only for Father’s, so I think part of the problem is also. Uh, in a more initial part of it, most of the activities, most of the information are always targeting mothers and directing personally to mothers. So...if you are a father and if you are willing to be part of it, you are going to feel that you are not part of that.

HCI researcher

The same is true for grandmothers with only 4 articles including grandparents [89,144,151,152], even though the impact of grandmothers on research activities is well documented. Scott et al [144] directly mentioned the negative impact of not including grandmothers in their study. They are supported by Mushaphi et al [152], who reported similar findings. Both author groups now advocate for the inclusion of grandmothers, as they have a direct impact on the success of MCH interventions and studies. The review further included 16 articles that referenced single parents in areas such as nutrition [51,52,60,85,90,91,144,152,153] and maternal HIV [52,92]. Finally, 2 articles referenced unspecified caregivers [78,90].

The overwhelming presence of CHWs identified as caregivers is prevalent in all disciplines, whereas the public health sciences provided the most varied list of caregivers; for example, most of the articles that included fathers (n=7), single parents (n=8), and other caregivers such as grandparents.

### Research Outcomes Investigated in the Current Literature

Nutrition was measured in 14 studies in which the interventions predominantly targeted the promotion of and information regarding EBF for ≥3 months (n=8) [26,52,66,80,84,85,146,152]. This is followed by a focus on the donation of breast milk (n=2) [56,93], maternal well-being while breastfeeding (n=1) [94], stunting (n=1) [53], general infant and young child feeding (n=2) [66], and finally, a focus on including fathers in their children’s nutrition and breastfeeding (n=1) [86]. The scoping review further highlighted that cultural and religious beliefs and misinformation often undermine EBF and other nutritional outcomes [152]. Using platforms such as SMS text messages framed around the benefits and self-efficacy of breastfeeding [52] improves parents’ awareness and information regarding breastfeeding [93]. Voice messages that assist with feeding information [66], video-based entertainment-education implementations that are simply and carefully constructed in the language and context of the target community [78], and chatbots that share information on breastfeeding practices [80] have been successfully implemented to provide factual and accurate information to mothers. However, the literature also highlights that implementing technology alone will likely not have the desired impact as community leaders and older adults [144] should not be underestimated. Community-based programs tailored to their target communities, which include older adults [144], CHWs [80], local celebrities [78], and government initiatives [91] aimed at promoting EBF and good nutritional practices, have proven to be very successful. Therefore, it is clear that these stakeholders need to be included in any digital intervention aimed at the communities to which they belong.

The dominance of measuring nutrition is prevalent in the public health sciences, with this discipline accounting for 11 articles related to nutrition. However, this is further evident in our interview data, with nutrition serving as a measurement across disciplines with 7 of the researchers and practitioners, including nutrition and infant and young child feeding as areas that they have studied or are studying. The researchers provided the following comments:

It would be good not to have to go to the health Center to get some of the messages we get about infant feeding. So, thinking in both of those contexts, the idea of message sharing with mothers through, you know, mobile. Devices in some ways is something we’ve thought about.

Public health researcher

I’m currently working on a project in Peru which is. Maternal and child nutrition and when we reach probably sometime next year will include a co-design element to it so we are working towards Co designing interventions.

Practitioner

The literature measures child development to a lesser extent, with 11 studies mentioning it. The vast majority of this literature focuses on the use of mHealth tools in the form of a mobile apps or mobile-friendly web-based applications to aid child development (n=8) in areas such as parent evaluation developmental screening tools [82], vision and hearing screening [47,95], computer games aimed at children with fetal alcohol syndrome [86], and technologies aimed at behavioral change to increase child development [154]. There is also a focus on how technology can be leveraged to support child health and development [27]. The adoption of screening tools such as Ages and Stages [,^155^] and Parents Evaluation Developmental Screening apps [82] provides better services, access to information for decision-making, and record keeping in early childhood development. The literature further indicates that LMIC face many challenges in measuring and supporting child development, particularly in rural areas [27,82]. These areas provide further complexities in terms of available infrastructure, electricity, and cellular coverage [27], which has hindered the deployment of digital-based initiatives aimed at these communities. The role of CHWs is highlighted as a crucial support mechanism for overcoming the lack of trained and available medical staff in LMIC. Thus, many of the deployed studies inquired about the acceptability [74], sustainability, and usability [82,95] of apps from the perspective of these CHWs.

The public health sciences accounts for the most articles related to child development (n=6), followed by HCI in health research (n=4), and finally the social sciences applied to health (n=1).

Our interview data support the fact that child development is studied to a lesser extent than other health concerns and nutrition. Early childhood development is an important part of any child’s development, and more work is needed in this area [156]. In our network, 2 researchers from social sciences applied to health and only 1 public health sciences researcher researched this area:

We’re working with the Maasai community who are semi nomadic community and in that project we’re looking at early child development and we’re trying to identify things that the community already do well to support early child development.

Public health researcher

And my work focuses on children is on their eating behaviours in particularly, but also their kind of activity behaviours and their early childhood development as well.

Social sciences applied to health researcher

The review identified a further 13 studies relating to a variety of health outcomes including the prevention of HIV mother-to-child transmission (n=7) [14,15,58,150,152,157,158]; monitoring and managing antiretroviral treatment (ART; n=2) [157,158]; encouraging and tracking timely immunizations (n=1) [64]; the management of smoking and alcohol consumption (n=1) [96]; the use of technology to enable remote telemedicine to address the lack of trained medical staff (n=1) [97]; and finally, to provide health education to address the high infant and mother mortality rates in LMIC (n=1) [97]. The management, monitoring, and prevention of HIV are prevalent, with little work done on areas such as the general health and well-being of mothers and children.

The literature highlights the following digital interventions: (1) nurse-delivered mobile phone ART counseling [158]; (2) SMS text message and voice reminders about ARTs [158]; and (3) mobile-assisted personal interviews to gather information regarding the prevention of HIV mother-to-child transmission, HIV prevalence, and ART have high levels of acceptability as successful and sustainable interventions [58]. However, these interventions also raise concerns about privacy and the accidental disclosure of HIV status. Digital technologies further enable better monitoring and reporting of HIV prevalence and serve as a good conversation starter, which in turn highlights the visibility of the medical issue that the technology hopes to address [143]. Older and more robust technologies, such as nurse-based mobile phone ART counseling, can reach deep into rural areas and address communities with low literacy rates [98].

It is not surprising that the public health sciences accounts for most articles focusing on health outcomes by providing 8 articles [35,55,57,63,83,87,92,156] followed by the social sciences applied to health, which provided 2 articles [91,96], and the HCI in health research, which provided 1 article [158]. Unfortunately, the overwhelming amount of research into maternal health, nutrition, and child development has led to a lack of research on the use of digital interventions for general health outcomes, which could still have extreme consequences for MCH.

The lack of research on general health is also evident in the interview data, with researchers mentioning the following:

So, they did. When we did this nine-month project, they realize that they wouldn’t have much work to do in the space of pregnancy care because in those areas the public health workers are quite active, so that’s why they are. These health worker collectives are usually look at now. Non communicable diseases like diabetes and others hypertension. Whereas because the public health is not focused on that and driven to care.

Public health researcher

Maternal mental health is severely underrepresented, with only 5 articles representing this domain. Only 2 articles [62,64] contained a digital component in the form of passive sensor data derived from wearable technologies [62]. Unfortunately, this study [62] was set to start data collection in November 2019, and the findings are not yet available. The literature further indicates the importance of including maternal mental health in primary care packages [52,64] as the far-reaching effects of the mental health of mothers on child development [52], nutrition, hygiene [52], and cognitive development [99] are well known and have not been addressed in the South African context as well as in other LMIC. It is interesting to note that the public health sciences account for most of the articles (n=3) that focus on maternal mental health and not the social sciences applied to health; however, the inverse is true for the interview data, where only the social sciences applied to health account for participants conducting work in this area.

Our interview data support the fact that mental and maternal health are underrepresented, and it is an area that should be prioritized for future research. Two of the social scientists interviewed are currently focusing on the following projects, maternal and general mental wellness:

We’re working with the prison service there to look at digital technologies and mental health for the prisons in Guyana. So, we’re working with all five prisons there. That’s, I mean, that’s a very big project.

Social sciences applied to health researcher

So, we actually started to provide meals, but we began to realize the severity of mental illness and how pregnancy and childbirth exacerbates that.

Social sciences applied to health researcher

Usability studies and considerations are also underrepresented, with 3 articles explicitly addressing the usability of technology tools aimed at MCH. The literature highlights usability considerations in areas such as heart failure [100], digital and maternal storytelling [159], and preeclampsia triage [101]. HCI in health research accounts for all articles measuring usability.

Very few articles (n=4) measured the use of ICT deployments aimed at MCH; use was measured in terms of mobile apps [51] and SMS text messaging platforms [102]. Public health sciences account for all the articles measuring use.

## Discussion

### Overview

This scoping review shows that the current literature focuses more on CHWs than on the direct beneficiaries of digital MCH interventions. We further found that key players, such as fathers and grandparents, are often excluded as participants, even when the impact of these key players is important for the success of any intervention. Fathers often feel alienated and out of place when they are not specifically and carefully included in the research and design of interventions. Even though the researchers provided strong values such as trust, respect, and inclusion, the communities were still included too late in the research and design process. Researchers also framed designing with or for communities as very different, not only between disciplines but also within a single discipline. It is also evident that the advances in technology and more accessible and affordable smartphones and data have created opportunities for the use of custom-developed mobile apps and wearable technologies. Unfortunately, most work done in digital MCH still focuses on targeting the physical health of participants or their children, while there is a distinct and well-articulated need for more work in maternal mental health and well-being. Finally, the inability of research efforts in digital MCH to inform and affect the government and policy remains a major challenge. In the following section, we discuss our major findings in detail.

### Asking the Wrong People, the Wrong Questions Too Late

#### CHWs Versus Direct Beneficiaries

Our findings indicate that MCH research in LMIC and Africa mostly targets CHWs rather than the parents who are the direct beneficiaries of the planned interventions. This was prevalent not only in the articles but also in the interview data and across all disciplines. In addition, practitioners mostly focused on CHWs in most of their work.

Thus, CHWs are treated as proxies for the actual communities in question. This is because CHWs are key players, as they are often trusted and more likely to understand the communities that they serve and could play a pivotal role in the success of any digital intervention [160]. However, our findings indicate that CHWs are often only consulted after the design process or engaged during the design process, instead of being treated as equal design partners. Sanders and Stappers [34] explain that consulting with users does not equate to conducting co-design; here, the researchers must consider the fact that CHWs are expert users in the problem under investigation and should be involved from the start of the design process. This is further evident if we consider that *inclusion* is listed as the most common value across most disciplines; however, *listening* and *communication* are mentioned the least. This mismatch in values and the delayed involvement of CHWs and other caregivers in the design process go a long way to explain some of the current gaps in digital MCH.

In addition, CHW-based design has been a major focus in HCI research that supports day-to-day work and professional responsibilities of CHWs, which often means that CHWs are requested to use the designed app rather than being involved in the design process. Many of the studies included in our review highlight interventions aimed at assisting CHWs in providing health education [69] or digitizing immunization records [47,82,83]. CHW-based design does not often consider the lived experiences of parents and caregivers and will never truly expose what these parents genuinely need and want to use [161]. Kapuire et al [162] explain that communities have their own literature and their own concepts of knowledge, and these communities should be allowed to influence the shape of digital interventions that will honor their cultural identity. Researchers must engage directly with these communities to create interventions that represent their ways of doing, saying, and being [162] if they truly want to benefit them.

Thus, a greater focus on family-oriented design aimed at voluntary use [161] that benefits the family [163] is needed if the interventions are to be successfully implemented and their impact measured regarding their usefulness from their direct target audience. Unfortunately, only 1 paper [87] from our scoping review considered families and family structures in their design by focusing on the extended family. This trend is not new and is still present in HCI in the health research discipline, with Fails et al [163] explaining that the need for work in this area has been identified as early as 1992, yet there is a limited body of work focusing on this area. This is concerning, as CHWs cannot always act as proxies for the communities they work with, and the only way to address the MCH problems faced by these communities is to engage them directly.

Engaging with CHWs is also challenging because of the time and resources necessary to conduct co-design, which might make it hard for researchers and practitioners to include CHWs and parents from the beginning of the design process. Co-design workshops can be time-consuming and require consistent participation. Many family members will not be able to forego generating an income for the duration of a study, cannot guarantee consistent participation, and are often not able to participate in research activities that take part in scheduled time slots [163]. There is a need to ensure that resources are available to effectively engage community participants before, during, and after the design process to help build trust, relationships, and a sense of ownership.

#### Exclusion of Fathers and Other Caregivers

South Africa and other African countries have a high rate of absent fathers (fathers who do not play a role in raising their children or are not involved in supporting the maternal health of the child) [164]. This often leads to mothers relying on the immediate and extended family to help them raise their children. Thus, African countries do not fit into the notion of nuclear families [165]. African families are classified as families that include multiple caregivers [13] such as grandparents and other extended family members (aunts, uncles, brothers, and sisters) [166]. Not surprisingly, our findings indicate that fathers and other caregivers are largely excluded from designing and creating digital interventions aimed at MCH. Most of these 11 articles focused on nutrition as the key role of fathers in MCH in terms of helping to reduce the likelihood of preterm birth [167], supporting the development of healthy psychological well-being of mothers and babies [168], and supporting the general well-being of children as they grow into adults.

Although some studies, which mainly focus on nutrition, have been conducted to include fathers in digital MCH interventions, our interview data indicated that fathers further felt out of place when using digital MCH interventions that were not explicitly designed with and for them in mind and wished to be included in the design process from the start. Cosson and Graham [169] highlighted the fact that fathers are often left to feel like the third wheel or a secondary substitute caregiver rather than a primary caregiver for their partner and offspring. This was further evident in our findings, which indicated that fathers were mainly included when their children’s nutrition was considered. While there is an undertone of parental incompetence being unfairly applied to many more fathers than mothers [170], many fathers from the articles in the review stated that they are a crucial part of a parenting or pregnancy team and that the current lack of recognition often damages the role they play as fathers, limiting their engagement with parental support activities [169].

The same holds for the inclusion of other crucial caregivers, such as grandparents, with only 6 articles targeting or discussing the involvement of these caregivers. Our findings indicate that grandmothers are likely to impact the success of digital interventions aimed at mothers living in societies that follow patriarchal structures [171]. Grandparents provide advice, often guiding young mothers as they parent their infants [172]. The exclusion of these key players can influence the potential impact that digital health interventions and MCH programs can have in LMIC. Thus, technology aimed at the notion of nuclear families will not be a good fit for family structures that exist in the Global South. The cultural identities, differences, and realities of communities must be reflected in the interventions intended for them.

### The Methodological Discrepancies of Community Engagement Versus Co-design

There is a clear divergence in how practitioners and research participants from different disciplines frame the design with or for communities. This divergence exists not only between disciplines but also within disciplines, with each discipline having at least 1 participant who did not differentiate between community-based co-design and community engagement. Both the social sciences applied to health and public health sciences thus provided broader differentiation of the 2 concepts. However, the interviewed stakeholders did not equate community-based co-design as empowering the user as a co-designer and giving them agency in their context and often only included them to conduct usability studies after the intervention was created and, in some cases, after the intervention was deployed. These disciplines more likely refer to community engagement when they mention co-design. This methodological confusion is not unique to this study. The confusion among participatory design, co-design, and community engagement has existed in the HCI space for some time. Participatory design has a long history of attempting to enable an active role of multiple stakeholders in system design with the hope of better matching technology features with the user’s needs through the use of mutual learning and collaborative prototyping [173].

In relation to co-design, as defined by Sanders and Stappers [34], “The creativity of designers and people not trained in design, working together in the design process” [78], we see a similar confusion here with community engagement, as these 2 terms are closely related but not the same. Community engagement refers to including the community in some way, at any stage of the process. The ladder of participation by Arnstein [174] describes engagement as “doing for” the community as opposed to “doing with” the community. Community-based co-design aims to include the community by “doing with” [174] the community by reducing power differentials to increase equal participation among all stakeholders from the start of the design process. Thus, there are differences in how these disciplines interpret these concepts in practice.

We identified this methodological confusion predominantly in the public health and social sciences applied to health disciplines, but more work can be done to better understand and use co-design in all disciplines included in this study. In general, the review and interview data highlighted that research and practice in MCH often do not conduct co-design but, in some instances, conduct usability studies because the participants are not included as equal design partners from the grassroots of any project that aims to solve problems faced by these communities.

### A Congruence and Divergence of Values

Our findings indicate that all disciplines included in this study valued inclusion, trust, and respect when working with communities. In fact, inclusion is the most mentioned value in both the review and interview data. However, collaboration, communication, and understanding were mentioned least. These values are also at the core of the implementation of community-based co-design. There were also subtle differences in which values each discipline believed was the most important, this ranged from “benefits the community” for the HCI in health research to “beneficence” for the public health sciences and “inclusion” for both the social sciences applied to health and practitioners. An interdisciplinary approach to MCH research has the potential to combine all the values listed and discussed by each discipline to create a value framework that will, in turn, capture the true essence of community-based co-design. This framework can assist researchers new to community-based co-design and serve as a reminder of what we aim to achieve by using this research method.

The stakeholders interviewed as part of our consultation clearly showed that there is a strong set of values present in their work, which aligns with the values of sensitive design. Consider, for example, the words of Friedman et al [175], “the design of technology that accounts for human values in a principled and comprehensive manner*.*”

Later work by Friedman et al [175] focused on value-sensitive design methods that include value-oriented mock-up, prototype, or field deployment, which could help researchers interested in digital MCH honor these values by bringing them to the forefront of their work. A further recommendation could be to consider value-sensitive design as a starting point for co-design work aimed at communities.

### Opportunities and Key Threats for Technology

The GSMA State of Mobile Internet Connectivity Report [176] explained that smartphones are becoming more affordable, leading to increased smartphone use worldwide. LMIC see a 28% annual growth in smartphone use. The report further states that mobile internet is becoming more relevant in LMIC as data are becoming more accessible and affordable. Finally, there is an increase in the number of LMIC that develop their own mobile content. These countries are designing mobile content based on their cultures, realities, and lives, rather than subscribing to mobile apps from other developing nations. Our findings mirror this report by clearly showing the dominance of custom-developed mobile (mobile software written for a specific cause and not leveraging apps shipped with a mobile device) apps in the literature. Custom-developed apps provide researchers and practitioners with the option to create tailor-made interventions that can include images, video text, and voice to not only reach those who are digitally included but also cater to participants who are digitally excluded or lack digital and general literacy [177]. They can also reduce the costs involved with Unstructured Supplementary Data Services and SMS text messages [176] by using cache or locally stored information that does not involve further costs.

SMS text message and Unstructured Supplementary Data Services implementations are often preferred because of their low cost and reliability [44]. However, many studies have indicated that these implementations are costly to the provider and vulnerable to network conditions. The text-based nature of these apps further does little to support low-literacy users, and bulk SMS text messages are not sufficient to enable and maintain relationships between health professionals and their patients [159]. Finally, the broadcast nature of these messages creates an opportunity for providers to celebrate the number of subscriptions, instead of the number of messages read and interacted with. A recent study in South Africa indicated that as little as 8% of MomConnect users used the web-based helpdesk provided [178], and other studies [102,179] highlighted the fact that more research is needed on the outcomes of SMS text message–based health interventions.

Finally, our findings indicate that wearable and sensor technology has not reached large-scale investigation and deployment in the MCH arena in LMIC, and a further search, specifically focusing on sensors and wearable technologies in LMIC, produced only four additional articles that focused on (1) measuring the quality of sleep of pregnant mothers, (2) a sensor-enabled digital arm band to measure health statistics for preeclampsia, (3) wireless skin sensors to monitor physiological data in the NICU, and (4) measuring nutrition in LMIC [180-183]. Other areas of health research in LMIC show a trend similar to these studies, including sensor and wearable technologies for remote patient monitoring [184], monitoring optimal health and nutrition [185], and measuring sepsis in patients in the emergency room [186]. This is unfortunate because these technologies do not always have to carry high costs and could be leveraged to provide innovative solutions to the current MCH challenges in LMIC.

Aggarwal and Jagtap [187] explained that digital and wearable technology reduces the need for manual intervention from the user because it negates the need to read SMS text messages or open apps. They further explained that the process of data gathering is simplified and automated, is less likely to contain errors, and has the potential to revolutionize digital health care [186]. Finally, a study in South Africa explored the use of wearable technologies and cloud-based services to reduce the need for in-person consultations, which could alleviate the burden of the lack of medical resources in these countries by removing the need for health workers to physically and manually gather data needed to make diagnoses or support patients [62]. It is important to note that the cultural nuances present in the aforementioned technologies still exist when deploying wearable technologies. Kohrt et al [63] explained that researchers should take time to first establish and understand the cultural norms present in any community before they attempt to deploy wearable technology for passive data collection.

Our findings further showed that all disciplines and practitioners made use of the same technologies in completely different yet useful ways. More interdisciplinary work needs to be done so that the different disciplines currently investigating digital MCH can learn from each other and start to build best practice frameworks and knowledge bases for research that will follow in their footsteps.

### Well-being Versus Health in the Context of MCH

The articles in this review predominantly focus on physical health–related issues, with a much smaller focus on mental health and well-being. Only 5 (3.5%) of the 141 articles reviewed focused on maternal mental health. The importance of mental health was evident in the social and public health sciences in the review and was highlighted in our interviews, as they had a more prominent focus on mental health. Mental well-being was also more evident in the interviews than in the review and was also strongly represented in the public health sciences. Unfortunately, a very limited representation in the literature is not sufficient to address the current needs for mental health support, especially for new mothers.

The United Nation’s sustainable development goals have 1 specific goal aimed at health, which reads “Good Health and Well-being” with one of its targets listed as follows: “Reduce by one-third premature mortality from noncommunicable diseases through prevention and treatment and promote mental health and well-being*.*”

This sustainable development goal considers the physical health and well-being of humanity, which includes mental health; these are not seen as separate areas of concern but rather as a single entity. Graham et al [188] explained that it is nearly impossible to achieve good physical health without good mental health. The current blend of available articles and technologies addresses many physical needs but falls short in addressing the very real mental well-being needs of mothers.

This is echoed by many other authors who explain that mental health is a serious health challenge that needs to be addressed [189], particularly in the Global South, where there is still a large stigma and cultural influence on mothers accessing help for mental health–related issues [170,189-195]. Any technology aimed at addressing the much-needed gap that currently exists in terms of mental well-being will need to carefully navigate these stigmas to gain useful uptake and use. Some interventions that aim to address mental health already exist, such as advanced video communication to alleviate stress and improve a sense of connectedness [191] as well as ambient technologies in the form of a mood plant that tracks the mood of friends. This ambient technology also allows other friends to interact with the mood plant to reach out to friends who may need it [196]. Unfortunately, these apps are some of the few examples of work done to support mental health in the Global South, particularly in Africa. However, Blandford et al [103] highlighted the important fact that we need to consider that the stigma still attached to mental health; the limitations imposed by medical aid (for those who have it); and a genuine need for privacy, security, and trust from the possible users of such apps could all be reasons why this area of MCH is not experiencing similar growth as compared with other digital MCH initiatives [197,198]. These authors further explain that it is also crucially important to include mental health professionals, clinicians, and potential users in the design of such digital interventions.

We believe that community-based co-design that truly considers the stigma, needs for trust and privacy, and the understanding of cultural nuances executed in an interdisciplinary manner [199] could assist with the growth of digital interventions in this neglected yet incredibly important area of MCH.

### The Failure of Research in Implicating Policy

Both the scoping review and the interview data highlight a recurring gap between research and policy. Many researchers mention that their research fails to engage with policies that govern MCH in the countries where their studies took place. Uneke et al [200] explained that there is a direct need for implementation research, defined as scientific studies that change or improve government policies. Uneke et al [200] further noted that only 18 (3.8%) of the 471 articles sampled in their literature review ultimately improved government policy in terms of MCH. Vargas et al [201] explained that it is difficult to measure the impact of research on policy and that it is often unclear how research is considered in policy-making decisions. The findings from the literature can help inform and improve the livelihood and quality of life of citizens around the world when government policies are reviewed.

Perhaps making use of co-design combined with a cross-disciplinary approach could be beneficial for reaching and fostering involvement from policy makers. Specifically, using an approach that not only considers the cultures, norms, and needs of the communities but also tackles the problem from more than one angle will be more suitable. This could assist in creating digital interventions that can inform and improve government policy in terms of MCH in the Global South.

### Limitations

Our study has some limitations. First, we acknowledge that our consultation exercise purposefully included local and international stakeholders with expertise on MCH in many countries in the Global South (South Africa, Peru, India, Ghana, Malawi, etc) and beyond (United Kingdom, Portugal, United States, and Sweden). While this is an optional stage in the Arksey and O’Malley [40] framework for scoping reviews, we found this stage important to help us frame not only the scoping review but also the future agenda of our COMACH project and network, as previously suggested by Tricco et al [42]. The interview participants provided us with their interdisciplinary experience conducting research in the Global South, an overview of the methods, and their genuine understanding of co-design and community-based co-design. Future stakeholder consultation for scoping reviews in this context could explicitly explore stakeholders beyond their project networks to determine if any additional expertise is missing. Second, many of our stakeholders come from a privileged standpoint (industry, academia, and NGOs) and, besides their experience with Global South communities, they are not the direct beneficiaries of digital maternal health solutions. We did not include community participants in the stakeholder consultation due to the COVID-19 pandemic lockdown restrictions and data connectivity issues in low-resource settings. Community members are experts in their own living experiences, and we engaged with 4 geographically distributed communities in South Africa after the scoping review was conducted through interviews [198,202] and co-design activities [198] that complemented stakeholder consultations with interdisciplinary researchers and practitioners. Future reviews should aim to include community participants during stakeholder consultation to broaden their participation in the early stages of research and account for their local expertise, even if only within a small number of community participants. Third, we acknowledge that language also constrains the scope of the review, and future reviews should account for additional literature in which English is not the primary language.

### Conclusions

Digital interventions and MCH-based digital interventions have grown steadily in LMIC. Unfortunately, these interventions do not fully understand the community context and mostly consult community members after the intervention has already been designed and deployed. This lack of timely involvement of community members in a democratic and empowering manner [195], which has now become the standard for co-design and community-based co-design, has led to many digital interventions, excluding crucial key players who could directly impact the success of the intervention. A misalignment of what the community needs, the exclusion of important health considerations such as mental well-being, a failure to inform and influence policy, and a lack of innovation in the technologies implemented to support MCH in these communities are some of the major challenges identified. Fathers and other crucial caregivers are often not included in the design process, often resulting in their voices and experiences being absent in the design of digital interventions meant to serve their families.

Finally, there is a lack of common language among researchers working in community-based co-design, which often leads to systemic failures of ICT deployments aimed at digital and MCH interventions in LMIC. As researchers, practitioners, and designers of digital health interventions, we need to engage with different disciplines and truly involve the communities in the design of their own solutions if we want the technology to better support caregivers and generate any impact that can inform and influence policy makers to help us improve MCH in the Global South.

## Appendix

Multimedia Appendix 1 Search terms used.

Multimedia Appendix 2 Scoping review table and paper summaries.

Multimedia Appendix 3 Stakeholder demographics.

Multimedia Appendix 4 Interview guide.

## Abbreviations

ART: antiretroviral treatment
CHW: community health worker
COMACH: Co-designing Community-Based Information and Communication Technology Interventions to Enhance Maternal and Child Health in South Africa
EBF: exclusive breastfeeding
HCI: human-computer interaction
ICT: information and communication technology
LMIC: low- and middle-income countries
MCH: maternal and child health
mHealth: mobile health
NGO: nongovernmental organization
NICU: neonatal intensive care unit
PRISMA: Preferred Reporting Items for Systematic Reviews and Meta-Analyses

## Data Availability

The interview data set from the stakeholder consultation generated and analyzed during this study are not publicly available due to ethical concerns.
