# Supplementary material for: Digital Health Technologies for Maternal and Child Health in Africa and Other Low- and Middle-Income Countries: Cross-disciplinary Scoping Review With Stakeholder Consultation
Source: J Med Internet Res. 2023 Apr 7;25:e42161. doi: 10.2196/42161 (PMC10131761; doi:10.2196/42161)
Supplement: Multimedia Appendix 1 [file jmir_v25i1e42161_app1.docx]

Multimedia Appendix 1: Keywords and search terms used

| **Mother related search terms** | Mother, parent, mom, nursing mother, nursing, Maternity paternity, parental, postnatal, postpartum, Antenatal, prenatal, paternity, parental, caregiver |
| --- | --- |
| **Child-related search terms** | Infant, baby, foetal, toddler, Early childhood development, First thousand days |
| **Health and wellbeing related search terms** | Maternal and child Health, Health, well-being, Health, well-being |
| **Digital and m-health search terms.** | Digital Maternal and Child Health, Digital Maternal and Child Health Low-Income Countries, Mhealth + maternal and Child health, Maternal Health and technology, Child Health and technology, Mhealth women, Mhealth children, Mhealth pregnancy, Mhealth antenatal |

Maternal and child health digital LMIC country

Mother child digital LMIC country

Mother and child health digital Africa

Maternal and child health digital Africa

Maternal mhleath LMIC country

Mhealth Mother and Child LMIC countries

Maternal and child health with technology

Mother and child health with technology

HCI mother and child health technology Africa

Usability mother and child health , digital LMIC countries

Mother and child health app Africa

Mother and child health app LMIC countries
