# Supplementary material for: Digital Health Technologies for Maternal and Child Health in Africa and Other Low- and Middle-Income Countries: Cross-disciplinary Scoping Review With Stakeholder Consultation
Source: J Med Internet Res. 2023 Apr 7;25:e42161. doi: 10.2196/42161 (PMC10131761; doi:10.2196/42161)
Supplement: Multimedia Appendix 3 [file jmir_v25i1e42161_app3.docx]

Theme: Social Sciences Applied to Health

| **ID** | **Gender** | **Residing Country and City** |
| --- | --- | --- |
| SS01 | F | Cape Town, South Africa |
| SS02 | F | Loughborough, UK |
| SS03 | F | [Cardiff,](mailto:MannayDI@cardiff.ac.uk) UK |
| SS04 | F | Leicester, UK |
| SS05 | F | [Cape](mailto:fiona.ross@uct.ac.za) Town, South Africa |
| SS06 | F | Leicester, UK |
|  |  |  |

Theme: Public Health and Medical Sciences

| **ID** | **Gender** | **Residing Country and City** |
| --- | --- | --- |
| HM01 | F | Loughborough, UK |
| HM02 | F | Cape Town, South Africa |
| HM03 | M | Cape Town, South Africa |
| HM04 | M | Johannesburg, South Africa |
| HM05 | F | Loughborough, UK |
| HM06 | F | Polokwane, South Africa |
| HM07 | M | Hilton, South Africa |

Theme: Human Computer Interaction

| **ID** | **Gender** | **Residing Country and City** |
| --- | --- | --- |
| CS02 | F | Cardiff, UK |
| CS03 | F | [Cape](mailto:mdensmore@cs.uct.ac.za)Town South Africa |
| CS04 | F | Cardiff, UK |
| CS05 | M | [Cardiff,](mailto:VerdezotoDiasN@cardiff.ac.uk) UK |
| CS06 | M | [Bangalore](mailto:naveen.bagalkot@manipal.edu) , India |
| CS07 | M | Portugal, Spain |
| CS09 | F | Portugal, Spain |
| CS10 | F | [teresa.almeida@umu.se](mailto:teresa.almeida@umu.se) |
| CS11 | F | [Cardiff](mailto:StawarzK@cardiff.ac.uk) , UK |

Theme: Practitioners

| **ID** | **Gender** | **Residing Country and City** |
| --- | --- | --- |
| PR01 | F | Midrand, South Africa |
| PR02 | F | Midrand, South Africa |
| PR03 | M | Cape Town, South Africa |
| PR06 | M | Hilton, South Africa |
| PR07 | F | Cape Town, South Africa |
| PR08 | F | Stanford, USA |
