# Supplementary material for: Digital Health Technologies for Maternal and Child Health in Africa and Other Low- and Middle-Income Countries: Cross-disciplinary Scoping Review With Stakeholder Consultation
Source: J Med Internet Res. 2023 Apr 7;25:e42161. doi: 10.2196/42161 (PMC10131761; doi:10.2196/42161)
Supplement: Multimedia Appendix 4 [file jmir_v25i1e42161_app4.docx]

# Final Questions for Interviews

Clarify scope for the interview:

- Related to maternal and child health or well-being
- Specifically, first thousand days (conception to 2 years)
- Preferably with a digital component OR a community-based component
- CHWs are in scope
- Seeking references and projects especially in the following areas
- Other caregivers
- Community-based interventions

**Script Begins Here**

Hello. Thank you for meeting with me. My name is X and I am a [title] at [Organization]. The purpose of today’s interview is to help inform the literature and scoping review for the project. Co-Designing Community-based ICTs Interventions for Maternal and Child Health in South Africa (CoMaCH) is a project recognizing that community members are rarely empowered to voice their own priorities and actively participate in the design of interventions intended to benefit them.

For the purposes of analysis with our team, I would like to record this interview. Do I have your permission to record?

[If yes, start recording. If no, take good notes! Thank them either way.]

We are seeking to find out how people within the CoMaCH network understand “community-based co-design” as framed by the project, and to identify related literature and projects. The interview will compromise of six sections. First, we will start with definitions and values regarding community engagement and co-design, followed by projects in the field of MCH that you are currently involved in or know about, challenges of doing MCH projects due to the constraints of COVID-19, research design, suggested literature and projects related to MCH, and finally we will discuss how you envision yourself contributing to this project.

**Section 1: Definitions and Values**

## Definitions and Values (Please look or (share screen) at the Group Map on community engagement and/or co-design)


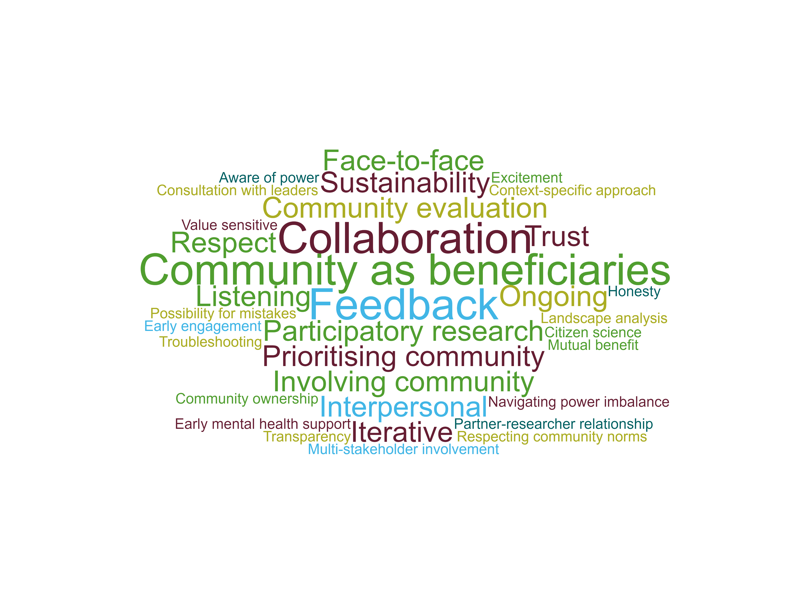


1. What do you think the CoMaCH project is about?
2. What do you understand by community-based and co-design?
3. What values do you attach to “co-design”?
4. What values do you attach to “community-based”?
5. How would you distinguish between “community engagement” and “community-based co-design" (if at all)?

**Section 2: Projects**

1. Please tell me about any of your projects that are related to CoMaCH which include the following:

- co-design,
- use of ICTs,
- or are community-based for MCH.

1. What role has technology (ICTs) played in these projects?
2. What role have communities played in these projects?
3. What are some of the challenges you have faced in carrying out your activities?

Follow-up Q: What challenges have you faced when engaging with communities/community members?

**Section 3: Challenges of doing MCH projects during covid-19**

1. What role has technology (ICTs) played in how you have adjusted (in your research activities) to the constraints due to COVID-19?
2. What are some of the challenges you have faced in carrying out your(research) activities? Follow-up Q: What challenges have you faced when engaging with communities/community members?
3. What new insights have you learnt over the past 6 months as you’ve adapted to the pandemic?
4. What are the sensitivities you would highlight to bear in mind when engaging and designing with parents/caregivers and healthcare workers in the context of MCH and COVID-19?

**Section 4: Research Design**

1. What is the best way to recruit and interact with community participants for interviews (both in normal circumstances, and under current social distancing restrictions)?
2. What suggestions do you have for enabling community participants to engage in our workshops (both in normal circumstances, and under current social distancing restrictions)?
3. What methods have been most successful when engaging with community participants?
4. Have there been any approaches that haven’t worked before or during the current circumstances?

*Follow-up Q: How have you managed to use different methods in your activities?

1. What are appropriate techniques for engaging with community-based participants, especially considering limitations due to physical distancing (e.g. phone interviews or activity books)?

*Follow-up Q:  How has existing power dynamics impacted research activities, if at all, in your experience? What are ways in which power dynamics can be addressed when engaging community participants?

1. What are some ethical issues that we should address and ask about in interviews and workshops?

**Section 5: Suggested Literature and Projects**

1. What journals, conferences or other resources do you think we should be searching to find literature and practice related to this project?

- Ask first
- Show them the list
- Ask about anything that was missing, and if there’s any resources that might be better than others or should be removed

1. This is the list of papers/projects/resources we found by you (or your group), what did we miss?

*Show them the list of their papers

1. What published papers or other resources (not necessarily yours) do you think we should definitely cite in a scoping literature review for this project? Why are these resources important?
2. This is the list of related projects we’ve identified in Africa, are there any other projects you are aware of? We are looking for projects in the last five years that involve communities in the use of digital technologies to support maternal and child health and well-being. Preferably, they would involve the parents and caregivers, but projects working with community health workers or frontline workers that work directly with parents are also fine.

## **Section 6: Contribution**

1. How do you envision contributing to this project?
2. How do you envision the community members and other stakeholders benefiting from the CoMaCH network?
3. How do you see yourself or your organisation benefiting from the CoMaCH network?

*Remind them about the workshop, where we will be presenting some of the early findings from this phase.
